# (E)-2-Hexenal Combats Rice Sheath Blight Through Direct Pathogen Inhibition and Host Defense Reprogramming

**DOI:** 10.3390/plants15101581

**Published:** 2026-05-21

**Authors:** Wenyan Fan, Wenjuan Wang, Xinyan Liang, Liting Feng, Xinyi Lv, Jitong Li, Yiping Wang, Jinglan Liu

**Affiliations:** 1 College of Plant Protection, Yangzhou University, Yangzhou 225009, China; 2 Jiangsu Province Engineering Research Center of Green Pesticides, Yangzhou University, Yangzhou 225009, China

**Keywords:** (E)-2-hexenal, *Rhizoctonia solani*, rice sheath blight, transcriptome analysis

## Abstract

Volatile organic compounds (VOCs) have garnered substantial research interest in recent years due to their biodegradability, low toxicity, and potent antimicrobial properties against various plant pathogens. As a typical herbivore-induced plant volatile (HIPV) elicited by *Nilaparvata lugens* (Brown planthopper, BPH), (E)-2-hexenal has been identified as a promising natural antimicrobial agent. In this study, we investigated the protective potential of (E)-2-hexenal against *Rhizoctonia solani* (*R. solani*) in rice, focusing on both its direct antifungal activity and host-mediated defense mechanisms. In vitro antifungal assays demonstrated that treatment with 100 μL/mL (E)-2-hexenal resulted in a 91.07% inhibition of *R. solani* mycelial growth after 48 h. Scanning electron microscopy (SEM) observation and chitinase activity analysis revealed that (E)-2-hexenal suppressed fungal growth by disrupting the structural integrity of the pathogen cell wall. Furthermore, 100 μL/mL (E)-2-hexenal effectively conferred protection to detached rice leaves. Whole-plant inoculation assays confirmed that (E)-2-hexenal pretreatment significantly alleviated disease symptoms and triggered systemic resistance in rice plants. Physiological and biochemical analyses showed that (E)-2-hexenal treatment enhanced the activities of defense-related enzymes, elevated hydrogen peroxide (H_2_O_2_) levels, and promoted the accumulation of defensive metabolites in rice leaves. HPLC-MS quantification further revealed significant increases in the endogenous levels of jasmonic acid (JA) and salicylic acid (SA). Transcriptomic KEGG pathway enrichment analysis indicated that differentially expressed genes (DEGs) were mainly involved in alpha-linolenic acid metabolism, diterpenoid biosynthesis, phenylpropanoid biosynthesis, plant–pathogen interaction, and plant hormone signal transduction. Collectively, these results suggest that (E)-2-hexenal enhances rice resistance to sheath blight disease via a dual-action mechanism: direct inhibition of fungal development and activation of host immune responses. Our findings highlight the potential application of (E)-2-hexenal and other VOCs in developing eco-friendly strategies for sustainable rice disease management.

## 1. Introduction

Rice (*Oryza sativa*) represents a vital staple food globally [1], yet substantial annual losses in productivity occur due to fungal diseases [2]. Among these phytopathogens, rice sheath blight, caused by *Rhizoctonia solani* (*R. solani*), is a soil-borne fungal disease; this soil-borne disease poses a serious threat to rice production [3,4]. Currently, no immune or highly resistant rice varieties are available, so disease control relies primarily on chemical methods [4]. However, excessive use of chemical fungicides can lead to the development of resistant pathogens, posing risks to food safety and public health [5,6]. Therefore, developing highly effective, low-toxicity, and environmentally friendly control agents is crucial for the sustainable management of rice sheath blight. Notably, green bio-pesticides derived from natural antifungal compounds represent a promising solution. In recent years, plant-derived natural compounds, especially plant volatile organic compounds (VOCs), have gradually become a research area of significant interest in the biological control of plant diseases due to their remarkable antimicrobial activity [7].

Plants, constantly challenged by microbial and insect invasions, have evolved sophisticated defense response mechanisms over time to counter these biological threats. Plant defense responses are mediated by intricate signal regulatory networks, which involve phytohormones such as jasmonic acid (JA) and salicylic acid (SA), as well as VOCs. In response to biotic challenge and abiotic environmental perturbations, plants rapidly synthesize and emit volatile organic compounds, which function as signaling molecules to elicit intrinsic defense strategies [8,9,10,11]. Recent studies have substantiated the efficacy of VOCs in controlling plant pathogens, including fungi and bacteria, by exhibiting antifungal and antibacterial activities—most notably by inhibiting fungal spore germination [12]. The efficacy of VOCs has been demonstrated against *Botrytis cinerea* in *Arabidopsis thaliana* [13], *Phytophthora infestans* on potatoes [14], and *Fusarium graminearum* on wheat [15]. In addition to their direct inhibitory effects, another critical function of VOCs is to act as signaling molecules to induce systemic acquired resistance (SAR), enhancing plant defense against pathogenic bacteria [16,17,18,19,20]. Interestingly, pre-treatment of *A. thaliana* seedlings with (E)-2-hexenal, Z-3-hexenal or Z-3-hexenol exhibited reduced lesion sizes when infected with *B. cinerea*; among these, (E)-2-hexenal showed the strongest protective effect [21]. Notably, VOCs are biodegradable and leave no harmful residues on agricultural products [7]. Given these beneficial properties, VOCs have immense potential for pathogen management.

(E)-2-hexenal is not only an abundant VOC in plant leaves, but also an herbivore-induced plant volatile (HIPV) [22]. HIPVs are primarily chemical substances released by plants upon herbivore attack, serving to deter insect pests and protect both the emitting plant and its neighboring individuals. Studies have shown that (E)-2-hexenal has a broad spectrum of biological activities, including antifungal, antibacterial, anthelmintic, and the induction of plant disease resistance [23,24,25,26,27]. (E)-2-hexenal has been demonstrated to exhibit significant fungistatic activity, effectively inhibiting the growth of various pathogens, such as storage-associated fungi (e.g., *Aspergillus flavus*, *A. niger*), Fusarium species (e.g., *F. graminearum*, *F. oxysporum* f. sp. lycopersicum), and *Botrytis cinerea* [28]. Studies have also shown that (E)-2-hexenal enhances plant disease resistance through multiple mechanisms, including elevating the activity of defense-related enzymes, promoting the synthesis of defensive metabolites, inducing the expression of defense-related genes and activating key signaling pathways [29,30,31]. Collectively, these findings indicate that (E)-2-hexenal holds great promise as a biofungicide for sustainable disease management.

Current research indicates that phytohormones, such as SA, JA, and ethylene (ET), play pivotal roles in plant defense responses against pathogen stress [32,33]. A general principle derived from earlier research suggests that biotrophic pathogens are often susceptible to SA-dependent defenses, whereas necrotrophic pathogens are typically inhibited by JA/ET-mediated responses [34,35]. However, accumulating evidence suggests that, under certain conditions, these pathways may also function synergistically to coordinate enhance plant disease resistance. Consequently, the JA, SA, and ET pathways are interconnected through a complex network of synergistic and antagonistic interactions, forming the backbone of plant defense signaling. Additional phytohormones such as abscisic acid (ABA), auxins, and gibberellins (GAs) also modulate these responses. Notably, VOCs exhibit intricate crosstalk with phytohormone signaling pathways [36]. Phytohormone signaling pathways can modulate the biosynthesis and emission of VOCs [37,38]. More importantly, the importance of VOCs is further highlighted by their ability to modulate phytohormone signaling, thus disrupting phytohormone homeostasis and influencing the plant’s defense strategies against pathogens [39,40,41,42,43].

Research has demonstrated that reactive oxygen species (ROS) serve as crucial signaling molecules in plant stress responses and signal transduction [44]. Plants have evolved a sophisticated antioxidant defense system to scavenge excess ROS and maintain cellular redox homeostasis, which includes antioxidant enzymes such as superoxide dismutase (SOD), catalase (CAT), peroxidase (POD), and polyphenol oxidase (PPO) [45,46]. Significantly, VOCs may also participate in plant defense responses and SAR induction by modulating ROS signaling pathways. Experimental evidence has shown that VOCs produced by *Pseudomonas fluorescens* PDS1 and *Bacillus subtilis* KA9 significantly enhanced the activity of defense enzymes (including phenylalanine ammonia-lyase (PAL), POD, and SOD) in pepper (*Capsicum annuum*) plants, while upregulating the expression of antioxidant genes associated with plant defense, thereby inducing systemic resistance against *Ralstonia solanacearum* [16].

In agricultural ecosystems, pest infestation and pathogen infection frequently co-occur (e.g., aphid-mediated virus transmission and stem-boring insects exacerbating sheath blight incidence). HIPVs, serving as crucial signaling molecules in tripartite plant–pest–pathogen interactions, could advance integrated pest management (IPM)) strategies and provide molecular targets for breeding crops with dual resistance to disease and pest resistance. Recent technological advancements have led to significant progress in research on VOCs for pathogen suppression. Researchers are not only continuously identifying novel antimicrobial volatiles but also elucidating their mechanisms of action and potential agricultural applications. However, the specific rice HIPVs that inhibit *R. solani*, and their effective concentrations, remain unidentified. As one such HIPV, the role of (E)-2-hexenal in the context of disease control in rice plants remains largely unclear, and its potential to induce disease resistance in rice warrants investigation. Our research team previously screened six representative HIPVs emitted by pest-infested rice plants [47]. Building upon this foundation, our study assessed their ability to suppress the rice pathogen *R. solani*. Our findings revealed that (E)-2-hexenal could directly inhibit hyphal growth of *R. solani*. Subsequently, we focused on the antifungal and resistance-inducing activities of (E)-2-hexenal, to explore the application of (E)-2-hexenal in the biocontrol of rice sheath blight, to elucidate the mechanism of action of (E)-2-hexenal and to facilitate the development of novel biocontrol strategies.

## 2. Materials and Methods

### 2.1. Test Materials

#### 2.1.1. Test Strain

The *R. solani* Kühn strain, belonging to the AG1-IA mycelium fusion group and a causal agent of rice sheath blight, was provided by Professor Chen Xijun from the Plant Pathology Lab at Yangzhou University.

#### 2.1.2. Preparation of Potato Dextrose Agar

Potato dextrose agar (PDA) was prepared using a standard formulation: 200 g of potato, 20 g of dextrose (Beijing Solarbio Technology Co., Ltd., Beijing, China), and 20 g of agar (Beijing Solarbio Technology Co., Ltd., Beijing, China), dissolved in 1000 mL of distilled water, with the pH maintained at its natural level.

#### 2.1.3. Test Agents

As for the VOCs, all the reagents used in this study were purchased from Shanghai Meryer Biochemical Technology Co., Ltd. (Shanghai, China).

#### 2.1.4. Test Rice Plants

For plant growth experiments and those involving foliar pathogens, the rice cultivar Zhong Hua 11 (ZH11) was utilized. After 2 days of seed soaking, seeds were subjected to germination treatment in a growth chamber under a 16 h-light/8 h dark (26 ± 2 °C) photoperiod with a relative humidity of 70–80%. Subsequently, the germinated seeds were grown hydroponically until reaching the four-leaf and one-heart growth stage. The hydroponic nutrient solution formula was prepared with reference to Yoshida [48].

### 2.2. Effects of VOCs on Hyphal Growth of R. solani

**Figure 1:**
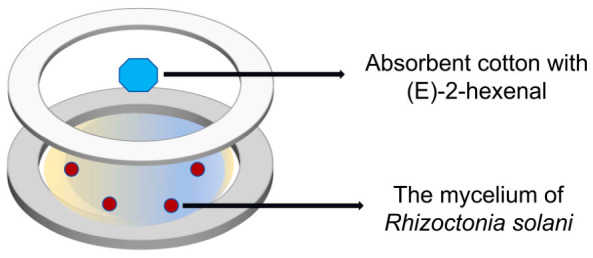
Schematic representation of in vitro VOC exposure.

The experimental method was adapted from a previous study [49]. The schematic diagram is shown as follows (Figure 1): The experimental setup consisted of a 90 mm Petri dish, with the bottom compartment containing the volatile matter device. A volatile dispenser was made by placing absorbent cotton into a small segment cut from a plastic tube cap. The VOCs were dissolved in hexane, mixed, and the solution (10 μL) was absorbed into the volatile matter device. The inner side of the lid contained PDA, and the pathogen mycelium was inoculated at the center. Parafilm was used to seal the Petri dish, ensuring that the setup prevents physical contact between the volatile matter device and the pathogens while guaranteeing sufficient airspace for the pathogens to be exposed exclusively to the VOCs emitted. The Petri dishes were incubated at 28 °C in the dark for observation.

### 2.3. Effects of VOCs on the Cell Wall Integrity of R. solani

#### 2.3.1. Preparation of Samples for Observation and Scanning Electron Microscope (SEM) Analysis

Colonies of *R. solani* were harvested from PDA and prepared for examination using GeminiSEM 300 field-emission electron microscopy (Carl Zeiss), as previously described in the literature and at the Yangzhou University Test Center. *R. solani* was cultivated on medium treated with different volatile compounds for 12 and 48 h. A 5 mm diameter section of PDA, taken from the edge of the mycelia, was placed in a glass Petri dish containing 2.5% glutaraldehyde fixing solution (10 mL) at 4°C. The fixing solution was then removed, and the samples were rinsed with sterile water three times for 10–15 min each. Dehydration was sequentially performed using ethanol solutions at concentrations of 30%, 50%, 70%, 80%, 90% and 95%, followed by a final dehydration with 100% ethanol solution, with each step lasting 10–15 min. Notably, the dehydration steps with 30%, 50% and 70% ethanol solution were conducted at 4°C. Finally, the samples were transferred to 100% ethanol solution containing Na_2_SO_4_, dried in a lyophilizer, and then imaged by SEM.

#### 2.3.2. Assessment of Chitinase Activity in *R. solani*

Hyphae were collected at 48 h post-treatment to evaluate chitinase activity in *R. solani*. The enzymatic activities were quantified using a commercially available assay kit (Beijing Solarbio Technology Co., Ltd., Beijing, China) in accordance with the manufacturer’s instructions.

### 2.4. Bioassay

#### 2.4.1. Detached Leaf Bioassay

A detached leaf bioassay was conducted using mycelia agar disks, following the method described in the literature [50]. Soaked facial tissues were placed on an iron plate, and fully expanded leaves (at 6-leaf stage) were cut into 20 cm segments and placed face up on the facial tissue. The surface of detached leaves was sprayed with (E)-2-hexenal solutions of different concentrations, while the control group was sprayed with water. The microenvironment’s relative humidity was kept at about 85%, and the setup was sealed with plastic wrap to retain moisture. A spherical mycelial agar block (5 mm diameter) from a 2-day-old culture of *R. solani* on PDA media was placed in the center of each leaf segment and incubated under a photoperiod of 14 h of light and 10 h of darkness at 28 °C. After incubation for 3 and 5 days, the symptoms were evaluated. Fifteen biological replicates were taken from each treatment group, and the development of symptoms was scored based on the proportional length of disease spots.

#### 2.4.2. Whole-Plant Bioassay

The effect of VOCs on the hyphal growth of *R. solani* was assessed using a hyphal growth rate assay. Once the mycelium of pathogens had fully colonized the Petri dish, typically within 48 h, a 5 mm diameter piece of PDA was excised and transferred to the center of a new Petri dish containing a volatile matter device. This device held different concentrations of VOCs, with each treatment replicated eight times. A Petri dish without a volatile matter device was used as a control.

VOCs were prepared in six distinct concentrations using 1 mL of hexane as solvent. Prior to treatment, (E)-2-hexenal was dissolved in hexane. (E)-2-hexenal is slightly soluble in water and readily soluble in organic solvents such as hexane, ethanol and diethyl ether. Hexane acts only as an inert solvent with no antifungal or eliciting activity itself. Therefore, the pure hexane blank control was set up to eliminate the interference caused by solvent volatilization and physical environmental factors, indicating that the experimental design is reasonable. Specific volumes of VOCs (1, 5, 10, 50, and 100 μL) were added for dissolution. After thorough mixing, the solution (10 μL) was absorbed into the volatile matter device. For esthetic presentation and ease of interpretation, the concentrations of the volatile substances were recorded as 0 (hexane control), 1, 5, 10, 50, and 100 μL/mL. In this study, the final concentration of the fungal suspension sprayed on plant leaves was adjusted to an OD_600_ value of 1.0. The colony diameter of *R. solani* was monitored on the lid of the Petri dish every 12 h by the cross method, with the plate diameter serving as a scale reference for the photographs. The percentage of pathogen suppression was calculated as follows: *R. solani* suppression (%) = (control colony diameter − treated colony diameter)/control colony diameter × 100%.

A whole-plant bioassay was conducted on rice plants, following the protocol described in the literature [3]. Then, 5 mm diameter PDA inoculated with *R. solani* was prepared as described in Section 2.4.1. Different concentrations of (E)-2-hexenal were applied to 4-leaf and 1-heart stage rice seedlings, ensuring all leaves were moistened. The control group was treated with sterile distilled water containing 0.1% Tween-80. After 12 h post-treatment, 5 mm diameter PDAs inoculated with *R. solani* were picked up with sterile tweezers and inoculated into the inner side of the leaf sheath, with one piece per plant, approximately 2 cm above the water line. Each treatment was applied to fifteen plants, and each test process was repeated three times to compile a comprehensive dataset. The plants were then covered with parafilm to maintain a humid environment conducive to infection. Rice growth was observed every day, disease symptom severity was evaluated at 10 days post-infection (DPI), and the disease index and control effect were calculated. Each treatment group comprising 15 inoculated rice plants was established as a discrete experimental unit. Disease severity assessment was conducted through quantitative evaluation of the lesion area percentage (LAP) and graded as follows: 0, absence of necrotic lesions; 1, LAP > 0% to ≤12.5%; 2, LAP > 12.5% to ≤25.0%; 3, LAP > 25.0% to ≤50.0%; 4, LAP > 50.0% to ≤75.0%; 5, LAP > 75.0% [51]. Both the disease index and the priming effect were calculated using the following formulas: Disease index = ∑ (number of disease plants at all levels × representative value at all levels)/(total number of investigated plants × the highest representative value) × 100; Priming effect (%) = (disease index of control group − disease index of treatment group)/Disease index of control group × 100%.

### 2.5. Enzymatic Antioxidant Assays

*R. solani* inoculation was performed after treatment with the optimal resistance concentration of 10 μL/mL (E)-2-hexenal as described in Section 2.4.2. Leaf samples were collected from infected rice plants at different time points for antioxidant assays and stored at −80°C.

#### 2.5.1. Assessment of Resistance-Related Enzyme Activities

Extraction of crude enzyme solution: Precisely weighed 0.1 g samples of the aforementioned rice leaves were homogenized individually in a mortar with 1 mL of ice-cold extraction solution. The homogenized solution was collected in a 2 mL centrifuge tube and centrifuged at 8000× *g* at 4°C for 10 min. The supernatant (crude enzyme solution) was pipetted into a new 2 mL centrifuge tube and placed on ice for testing.

To quantify the activities of antioxidant enzymes, peroxidase (POD), superoxide dismutase (SOD), catalase (CAT) and polyphenol oxidase (PPO), the above-described crude enzyme solution was subjected to analyses using commercial kits (Suzhou Comin Biotechnology Co., Ltd., product number: POD-1-Y, SOD-1-Y, CAT-1-Y, PPO-2-Y, respectively, Suzhou, China) according to the manufacturer’s instructions.

#### 2.5.2. Determination of Resistance-Related Substances

The hydrogen peroxide (H_2_O_2_) content was measured using a commercial assay kit (Suzhou Comin Biotechnology Co., Ltd., H_2_O_2_-1-Y, Suzhou, China), following the manufacturer’s guidelines. The level of lipid peroxidation was estimated by measuring malondialdehyde (MDA) content, a by-product of lipid peroxidation [52]. MDA content was quantified using a commercial assay kit (Suzhou Comin Biotechnology Co., Ltd., Suzhou, China), following the manufacturer’s guidelines. Determination of flavonoids and amino acids (AAs) referred to the instructions of the commercial kits (Suzhou Comin Biotechnology Co., Ltd., product number: LHT-1-G and AA-1-W, Suzhou, China).

### 2.6. Phytohormone Measurements

To investigate the effect of (E)-2-hexenal on phytohormone homeostasis, we quantified ABA, JA and SA content in leaf samples. Samples were obtained at various time points (0, 12, 24, and 48 h) post-infection, with three biological replicates per treatment and time point. The rice leaves were flash-frozen in liquid nitrogen and ground using a pestle and mortar. The ground leaves (100 mg) were employed for phytohormone extraction, following the procedures detailed in the literature [53]. Subsequent hormone measurements were conducted using HPLC-MS. ABA, JA, and SA contents in the treated and control groups were determined using the isotope internal standard method. According to the retention time of the isotope chromatogram, the peak area for each hormone was calculated. The hormone content was then determined by considering the peak area of the internal standard in the hormone isotope chromatogram and the initial sample mass.

### 2.7. Transcriptomic Analysis

#### 2.7.1. (E)-2-Hexenal Treatment and *R. solani* Inoculation

The experimental groups were designed as follows: CK: untreated non-inoculated control group; E: (E)-2-hexenal-treated (non-inoculated) group; Rs: *R. solani*-inoculated group; E_Rs: (E)-2-hexenal-treated inoculated treatment group. The rice in the artificial growth turnover box grew to the stage of 4 leaves and 1 heart. First, after spraying (E)-2-hexenal for 12 h, some rice leaves were taken, quickly frozen in liquid nitrogen and stored in an ultra-low-temperature refrigerator. Then, the other rice was inoculated. After 12 h of damage, the rice leaves were taken, quickly frozen in liquid nitrogen and stored in an ultra-low-temperature refrigerator. Each treatment was replicated three times. Leaf samples were harvested from rice plants and wrapped in aluminum foil, flash-frozen in liquid nitrogen, and subsequently stored in an ultra-low-temperature freezer at −80°C.

#### 2.7.2. RNA Library Construction and Transcriptome Sequencing

The rice samples were submitted to Shanghai Personal Biotechnology Co., Ltd. (Shanghai, China). for sequencing and RNA library construction. RNA library preparation and quality control were conducted as described previously [54]. After quality control, clean data reads were aligned with the reference genome (The Rice Annotation Project, IRGSP-1.0_genome.fasta) to generate mapped reads for subsequent transcript assembly and expression analysis. Multiple comparisons with the reference genome were made using Genescloud (accessed on 12 December 2024) (https://www.genescloud.cn/home). The number of fragments per kilobase of exon per million fragments (FPKM) was calculated for gene expression analysis. Differentially expressed genes (DEGs) were defined based on the following criteria: adjusted *p* value (*p*-adjust) ≤ 0.05, and default difference multiple ≥ 2. The Database for Annotation, Visualization and Integrated Discovery (DAVID) bioinformatics resource was used to analyze for over-representation of gene classes. Differential expression analysis was conducted using the gene expression levels across sample groups, and Gene Ontology (GO) functional annotation, as well as KEGG pathway enrichment analysis, was performed on identified DEGs.

#### 2.7.3. Identification of DEGs and Verification by qRT-PCR

DEGs were detected using DESeq2 2.0 software, with a threshold of absolute log2 fold change ≥ 1, and a false discovery rate (FDR) < 0.05 was used as the screening criteria. The fold change represents the ratio of expression among groups, and FDRs were obtained by correcting the significant *p* values. Six DEGs were selected for real-time quantitative PCR, and the accuracy of transcriptome sequencing was verified. HiScript ^®^Q RT SuperMix for qPCR (+ gDNA wiper) (Vazyme Biotech Co., Ltd., Nanjing, China) was used for reverse transcription, and Primer Premier 5.0 was used for primer design. Quantitative PCR was performed using the ChamQTM SYBR ^®^Color qPCR Master Mix Kit (Vazyme Biotech Co., Ltd., Nanjing, China). The mixed solution was added to 96-well plates, and the reaction mixture contained primers of the target gene (0.8 μL), 2 × ChamQ SYBR Color qPCR Master Mix (10 μL), and template (2 μL). cDNA from rice sheaths was used as template, ActinI was selected as the internal reference, and each sample was tested thrice. The relative expression of each gene was calculated by the 2^−ΔΔCt^ method [55].

### 2.8. Disease Resistance Assay of OsRbohB Overexpression and Knockout Mutant Plants

The methods for treating with (E)-2-hexenal, inoculating rice with *R. solani* and investigating the severity of the disease are the same as those in Section 2.4.2.

### 2.9. Statistical Analysis

Excel 2019 software was applied for data collation and GraphPad Prism 8 for graphing. Statistical significance between treatments was determined by analysis of one-way ANOVA. The difference was considered significant when the *p* value was < 0.05. Statistical analysis was carried out using Student’s *t*-test for comparison between two samples. The data were denoted as means ± SE and analyzed using SPSS 16.0 software.

## 3. Results

### 3.1. Suppression of R. solani by VOCs

**Figure 2:**
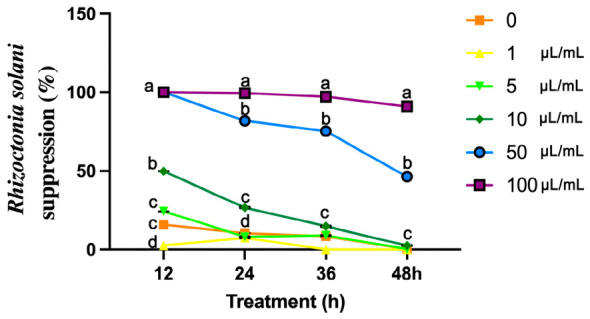
Suppression of *R. solani* by (E)-2-hexenal. The horizontal axis represents the treatment concentration. The suppression rate of (E)-2-hexenal on *R. solani* was quantified as the reduction in mycelial biomass compared to the control group. Data points represent means ± SE (N = 8). Different lowercase letters (a, b, c, d) indicate significant differences based on one-way ANOVA (*p* < 0.05).

To identify VOCs with inhibitory effects on *R. solani*, six representative VOCs were evaluated. The results revealed that (E)-2-hexenal exhibited the strongest inhibitory effect against *R. solani* compared to the other five treatments (linalool, leaf alcohol, 3-octanone, trans-caryophyllene, and α-pinene) (Supplementary Table S1). After 36 h of culture, the control group was almost completely covered by R. solani mycelium, while the group treated with 100 μL/mL (E)-2-hexenal showed negligible growth, suggesting that the minimum inhibitory concentration of (E)-2-hexenal to inhibit *R. solani* was 100 μL/mL (0.862 mmol/L). After (E)-2-hexenal treatment, concentrations below 10 μL/mL had little to no inhibitory effects on *R. solani* at 48 h. In contrast, at a concentration of 100 μL/mL, the inhibition rate of *R. solani* growth reached 91.07% (Figure 2).

### 3.2. Effects of (E)-2-Hexenal on the Cell Wall Integrity of R. solani

**Figure 3:**
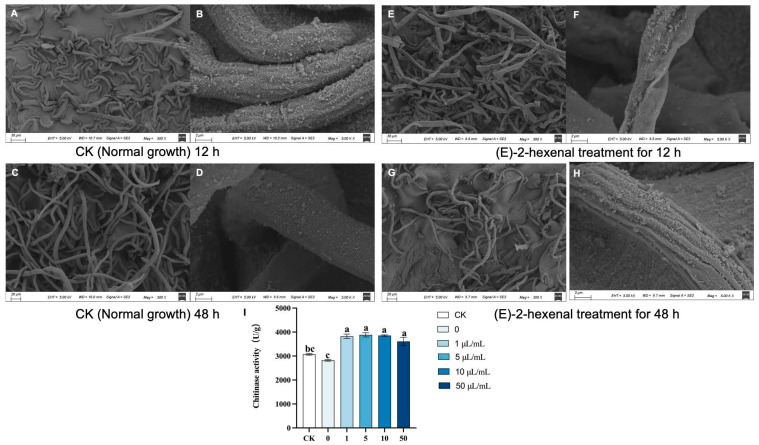
SEM images and chitinase activity of *R. solani* treated with (E)-2-hexenal. Note: (**A**) Control (normal growth) for 12 h, 300× magnification; (**B**) Control (normal growth) 12 h, 5000× magnification; (**C**) Control (normal growth) for 48 h, 300× magnification; (**D**) Control (normal growth) 48 h, 5000× magnification; (**E**) (E)-2-hexenal treatment for 12 h, 300× magnification; (**F**) (E)-2-hexenal treatment for 12 h, 5000× magnification; (**G**) (E)-2-hexenal treatment for 48 h, 300× magnification; (**H**) (E)-2-hexenal treatment for 48 h, 5000× magnification. (**I**) Effect of (E)-2-hexenal on chitinase activity in the cell wall of *R. solani*. Data points represent means ± SE (N = 3). Different lowercase letters (a, bc, c) indicate significant differences based on one-way ANOVA (*p* < 0.05).

The growth of fungal mycelium plays a crucial role in the pathogenicity of fungi. After (E)-2-hexenal treatment, the mycelial morphology of *R. solani* exhibited obvious alterations. The mycelium of the control group exhibited uniform thickness, smooth surface, and uniform thickness and even growth (Figure 3A–D). After 12 h of exposure to (E)-2-hexenal, the mycelium showed severe abnormal deformation, wrinkling, and abnormal cytoplasmic shrinkage (Figure 3E,F). After 48 h of (E)-2-hexenal treatment, the mycelium exhibited pronounced abnormalities, characterized by numerous folds on its surface, indicating severe disruption of cell wall integrity (Figure 3G,H).

Chitinase is an enzyme capable of hydrolyzing chitin in fungal cell walls, thereby inhibiting fungal growth [56]. Bacterial endophytes can degrade plant cell walls by secreting various carbohydrate-active enzymes (CAZymes), such as cellulases, xylanases, pectinases, and chitinase [57,58]. Chitinase activity in *R. solani* after (E)-2-hexenal treatment significantly increased compared to the control group (Figure 3I). The increased chitinase activity is consistent with the observed morphological damage, suggesting that the degradation of cell wall chitin may be one mechanism by which (E)-2-hexenal inhibits *R. solani* growth.

### 3.3. Protective Effects of (E)-2-Hexenal on Detached Leaves

**Figure 4:**
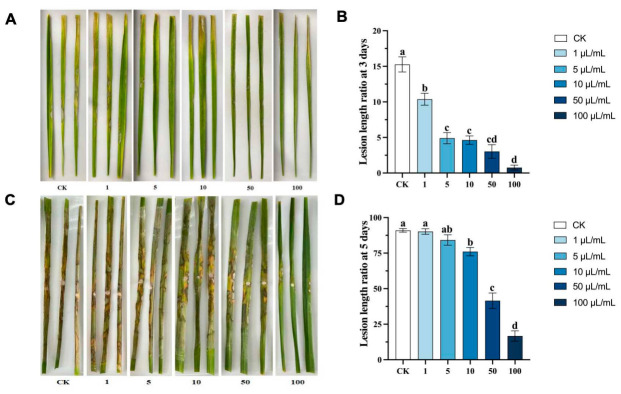
Protective effect of (E)-2-hexenal on detached rice leaves. Note: (**A**,**B**) Phenotypic changes and percentage of lesion length in detached rice leaves treated with (E)-2-hexenal at 3 DPI; (**C**,**D**) Phenotypic changes and percentage of lesion length in detached rice leaves treated with (E)-2-hexenal at 5 DPI. Data points represent means ± SE (N = 15). Different lowercase letters (a, ab, b, c, cd, d) indicate significant differences based on one-way ANOVA (*p* < 0.05).

Detached rice leaves were first treated with different concentrations of (E)-2-hexenal solution applied as a spray to the leaf surface and then inoculated with *R. solani*. At 3 days post-inoculation (DPI), necrotic spots were observed on untreated leaves and those treated with low concentrations of (E)-2-hexenal (Figure 4A,B). By 5 DPI, severe necrosis was evident in the control and low-concentration groups, while leaves treated with high concentrations (50 and 100 μL/mL) showed significantly fewer symptoms, with the control and low concentration groups exhibiting extensive necrosis. Notably, higher concentrations of (E)-2-hexenal corresponded to a reduced proportional length of disease lesions (Figure 4C,D). These results indicated that (E)-2-hexenal, at appropriate concentrations, confers significant protection to rice leaves, and preemptive application of (E)-2-hexenal on rice leaves can effectively inhibit the invasion and spread of *R. solani*.

### 3.4. Priming Effect of (E)-2-Hexenal on R. solani in Rice Seedlings

**Figure 5:**
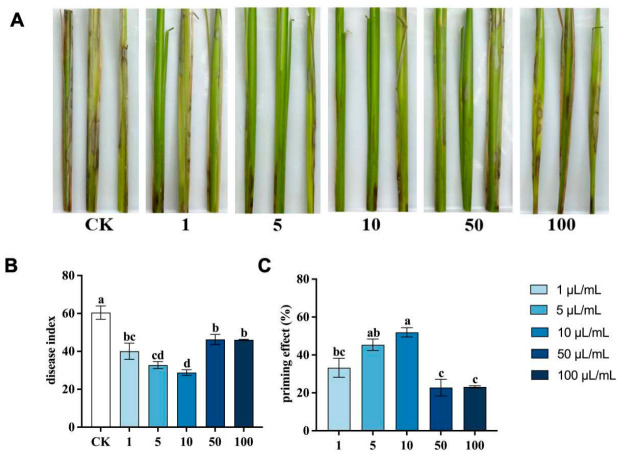
Priming effect of (E)-2-hexenal in rice seedlings at 10 DPI. Note: (**A**) Rice damage phenotype after inoculation with *R. solani* under different concentrations of (E)-2-hexenal treatment; (**B**,**C**) Disease index and priming effect of different concentrations of (E)-2-hexenal on *R. solani* in rice seedlings. Data points represent means ± SE (N = 15). Different lowercase letters (a, ab, b, bc, c, cd, d) indicate significant differences based on one-way ANOVA (*p* < 0.05).

The results of the whole-plant bioassay showed that (E)-2-hexenal treatment reduced the disease index of *R. solani*, especially with 10 μL/mL concentration lowering the disease index to 28.82 and demonstrating a priming effect of 51.93% at 10 DPI. Although the priming effect at 5 μL/mL was slightly lower (45.37%), the difference between these two concentrations was not statistically significant. However, at concentrations higher than 10 μL/mL, the priming effect decreased. These findings suggest that (E)-2-hexenal can induce rice resistance against *R. solani* (Figure 5).

### 3.5. Effects of (E)-2-Hexenal on the Rice Defense System

#### 3.5.1. Effect of (E)-2-Hexenal on the Activity of Defense Enzymes in Rice

**Figure 6:**
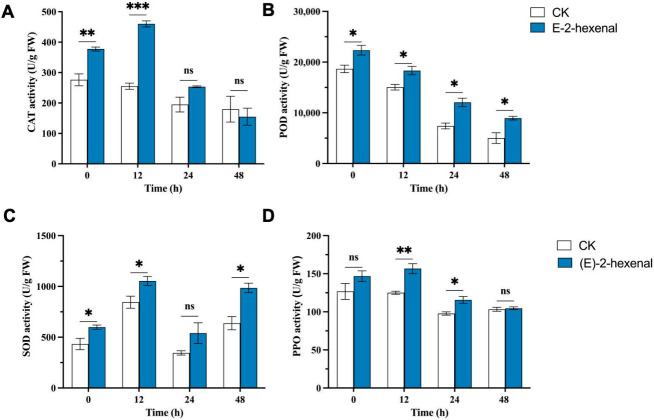
Changes in activities of (**A**) CAT, (**B**) POD, (**C**) SOD, and (**D**) PPO in (E)-2-hexenal treated rice leaves and untreated controls at different times after infection with *R. solani*. Note: Each bar represents the mean ± SE (N = 3). The asterisks indicate significant differences based on the Student’s *t*-test (* *p* < 0.05, ** *p* < 0.01 and *** *p* < 0.001; ns, not significant).

Antioxidant enzyme activities were increased in rice plants treated with (E)-2-hexenal following inoculation with *R. solani* compared to infected untreated plants, which may contribute to rice tolerance to oxidative stress. CAT activity in treated plants was significantly higher than in untreated plants, increasing by 36.60% compared to the infected control and 80.27% after 12 h post-inoculation (hpi) (Figure 6A). Following inoculation, although POD activity generally decreased following inoculation, it remained significantly higher in treated plants than in the infected control, reaching 1.79 times that of the CK group at 48 hpi (Figure 6B). SOD activity fluctuated over time. Treated plants exhibited significantly greater SOD activity than the infected control, with an increase of 54.41% at 48 hpi (Figure 6C). After (E)-2-hexenal treatment, PPO activity significantly increased by 25.28% compared to CK at 12 hpi and by 18.24% at 24 hpi (Figure 6D).

#### 3.5.2. Effect of (E)-2-Hexenal on the Content of Disease Resistance-Related Substances in Rice

**Figure 7:**
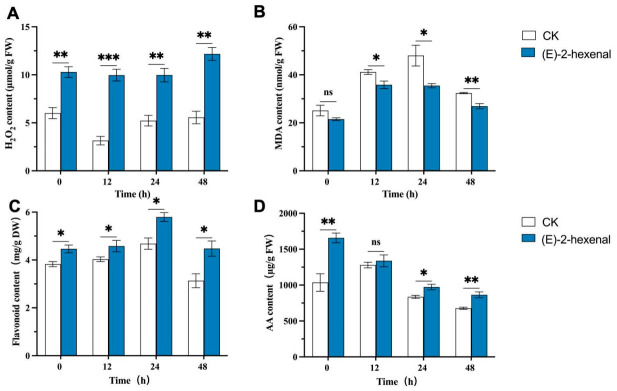
Changes in (**A**) H_2_O_2_, (**B**) MDA, (**C**) flavonoid, and (**D**) AA contents in (E)-2-hexenal treated rice leaves and untreated controls at different times after infection with *R. solani*. Note: Each bar represents the mean ± SE (N = 3). The asterisks indicate significant differences based on the Student’s *t*-test (* *p* < 0.05, ** *p* < 0.01 and *** *p* < 0.001; ns, not significant).

H_2_O_2_ levels were significantly elevated in (E)-2-hexenal-treated plants compared to untreated plants, peaking at 48 hpi, which was 71.43% higher than that of the CK group (Figure 7A). (E)-2-hexenal treatment significantly reduced the MDA content in rice plants. At 24 and 48 hpi, the MDA levels in treated plants were reduced by 26.11% and 16.87%, respectively, compared to untreated plants (Figure 7B).

(E)-2-hexenal treatment induced an accumulation of flavonoids in rice leaves. The content of flavonoids firstly increased and then decreased with time, reaching peak levels at 24 hpi with a 23.73% increase (Figure 7C). Similarly, the treatment induced an accumulation of ascorbic acid (AA) in non-inoculated leaves, with an overall increase of 29.11% relative to the CK. No significant difference in AA content was found between the treated and CK groups at 12 hpi. However, by 24 hpi and 48 hpi, the AA content in the treated group had increased by 10.37% and 27.56%, respectively (Figure 7D).

### 3.6. Effect of (E)-2-Hexenal on the Hormone Content in Rice

**Figure 8:**
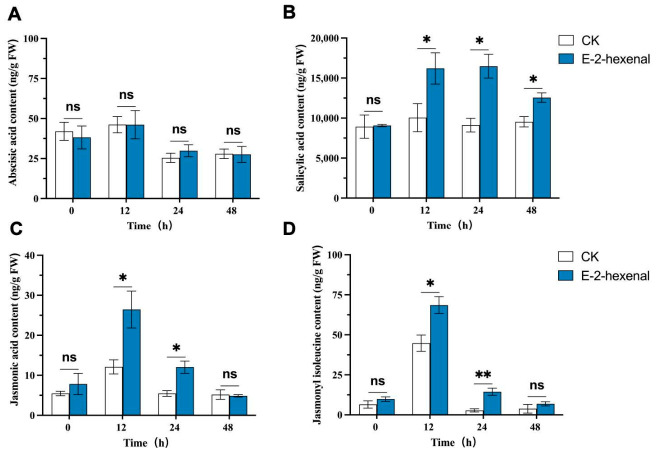
Effect of (E)-2-hexenal treatment on phytohormone levels in rice at different times after *R. solani* infection. Note: (**A**) ABA content; (**B**) SA content; (**C**) JA content; (**D**) JA-Ile content. ABA: abscisic acid; SA: salicylic acid; JA: jasmonic acid; JA-Ile: jasmonic acid-isoleucine. Data are mean ± SE (N = 3). The asterisks indicate significant differences based on the Student’s *t*-test (* *p* < 0.05 and ** *p* < 0.01; ns, not significant).

We performed HPLC-MS-based phytohormone profiling on rice plants exposed to (E)-2-hexenal. No consistent or significant effects on ABA levels were observed (Figure 8A). The content of SA showed significant differences after *R. solani* infection. At 12, 24, and 48 hpi, the (E)-2-hexenal treatment group increased by 61.23%, 80.79%, and 31.72% respectively compared to the control group (Figure 8B). The contents of JA and jasmonic acid-isoleucine (JA-Ile) showed similar trends, peaking at 12 hpi, with the content of JA in the (E)-2-hexenal treatment group increasing by 1.31-fold compared to the control group and the content of JA-Ile increasing by 53.12%. The contents of both JA and JA-Ile significantly decreased at 24 and 48 hpi, but (E)-2-hexenal treatment still resulted in significantly increased levels of JA and JA-Ile at 24 hpi, with increases of 1.20 and 4.22 times, respectively (Figure 8C,D). This demonstrated that exposure to (E)-2-hexenal indeed led to an increase in JA and SA in rice leaves.

### 3.7. Transcriptome Data

#### 3.7.1. Transcriptional Expression Changes in Response to (E)-2-Hexenal Exposure

Rice leaf samples were next subjected to a comprehensive transcriptomic analysis using the Personalbio Genescloud platform. After rigorous quality control and data cleanup, 8.94 to 15.75 billion clean reads were obtained by sequencing the cDNA libraries. Cluster analysis of DEGs was performed, a total of 3217 DEGs (1512 up- and 1705 down-regulated) were identified in the CK vs. E comparison group; a total of 6078 DEGs (2767 up- and 3311 down-regulated) were identified in the CK vs. Rs comparison group; a total of 7170 DEGs (3274 up- and 3896 down-regulated) were identified in the CK vs. E_Rs comparison group; a total of 2006 DEGs (1236 up- and 770 down-regulated) were identified in the Rs vs. E_Rs comparison group (Supplementary Table S2).

#### 3.7.2. Analysis of DEGs in Rice Inoculated with *R. solani* Under Different Treatments

##### GO Annotation of DEGs

**Figure 9:**
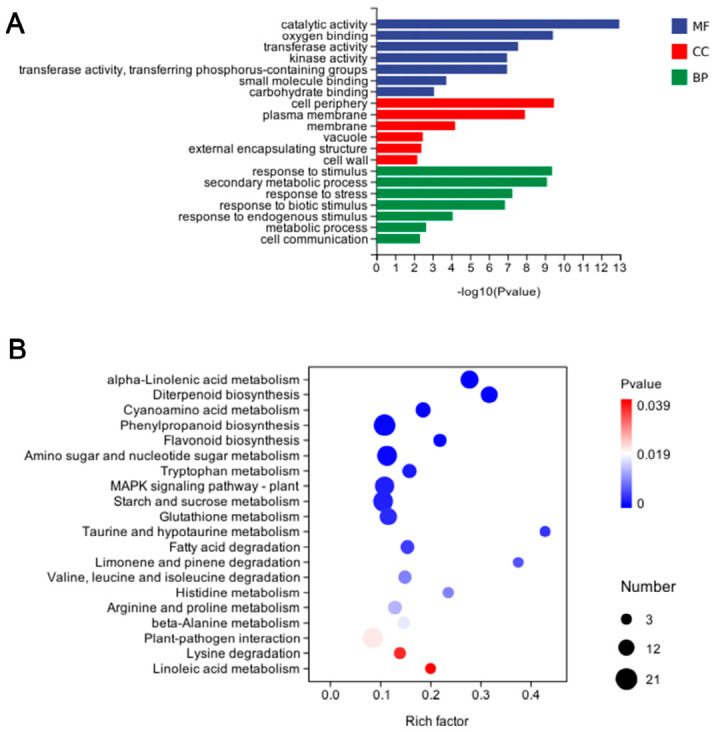
Overview of transcriptomic changes in rice induced by exogenous (E)-2-hexenal application and *R. solani* infection. Note: (**A**) GO annotation classification statistics of DEGs. The Y-axis represents GO Term, and the X-axis represents GO Term enriched −log10 (*p*-value); MF: Molecular function; CC: Cellular component; BP: Biological process. (**B**). Scatter plot of KEGG pathway enrichment of DEGs. The X-axis represents the rich factor (number of DEGs enriched in this pathway), and the Y-axis represents the KEGG pathway. The size of points indicates the number of differentially enriched (up- or down-regulated, related to the gene set selected during analysis) genes in the corresponding pathway, and the depth of colors indicates the level of significance. *p*-value: Enrichment significance *p*-value.

GO annotation was performed to investigate the major cellular functions of the DEGs in the Rs vs. E_Rs comparison group. GO analysis revealed that these DEGs were mainly enriched in categories such as response to stimulus, secondary metabolic process, response to stress, response to biotic stimulus, and metabolic process (Figure 9A). After (E)-2-hexenal treatment, the number of DEGs annotated to each GO function increased significantly. The number of DEGs annotated to metabolic process (GO:0008152) in the GO biological process was the highest, with 806 genes. The number of DEGs annotated to cellular anatomical entity (GO:0110165), cellular process (GO:0009987), catalytic activity (GO:0003824), and binding (GO:0005488) all exceeded 500 (Supplementary Table S3).

##### KEGG Enrichment Analysis of DEGs

KEGG analysis revealed that these DEGs were mainly enriched in several key pathways, including alpha-linolenic acid metabolism, diterpenoid biosynthesis, phenylpropanoid biosynthesis, flavonoid biosynthesis, MAPK signaling pathway-plant, plant–pathogen interaction and plant hormone signal transduction (Figure 9B). The highest number of DEGs was annotated to the phenylpropanoid biosynthesis pathway with 21, followed by plant–pathogen interaction pathway with 19, MAPK signaling pathway-plant with 17, plant hormone signal transduction pathway with 16 and alpha-linolenic acid metabolism pathway with 15 (Supplementary Table S4).

#### 3.7.3. Identification of DEGs and Verification

**Figure 10:**
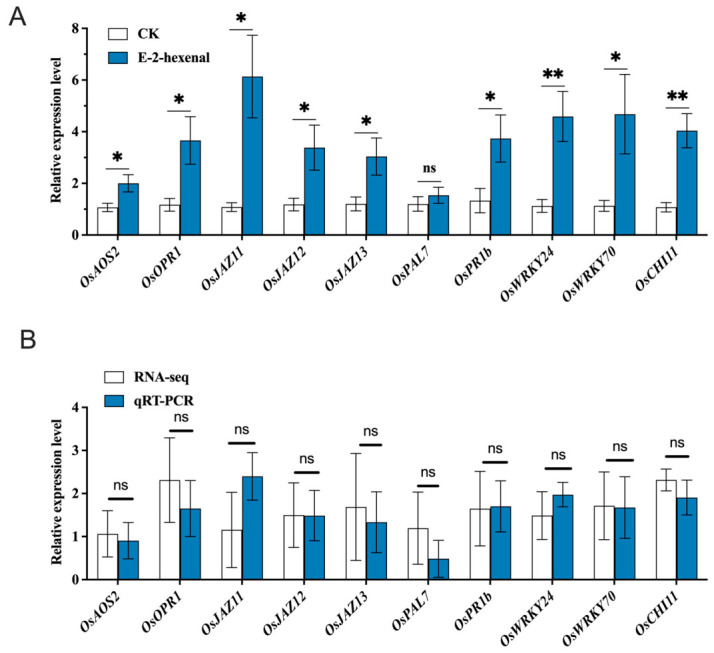
Validation of differential genes by RNA-seq and qPCR. Note: (**A**) Changes in gene expression. Each bar represents the mean ± SE (N = 3). The asterisks indicate significant differences based on Student’s *t*-test (* *p* < 0.05, ** *p* < 0.01 and ns, not significant). (**B**) qRT-PCR confirmation of genes is identified by transcriptome analysis. The relative expression level of each gene was expressed as the fold change relative to the control.

To verify the reliability of transcriptomic data, key genes exhibiting the most pronounced changes in JA and SA signaling pathways, along with genes in disease resistance-related pathways, were selected for qRT-PCR verification (Supplementary Table S5). The qRT-PCR analysis showed that the relative expression of genes treated with (E)-2-hexenal was higher than that of the control group (Figure 10A). The qRT-PCR results were consistent with the transcriptome sequencing results (Figure 10B), proving that the transcriptome data had a high reliability for further study.

#### 3.7.4. Functional Enrichment Analysis of DEGs

**Figure 11:**
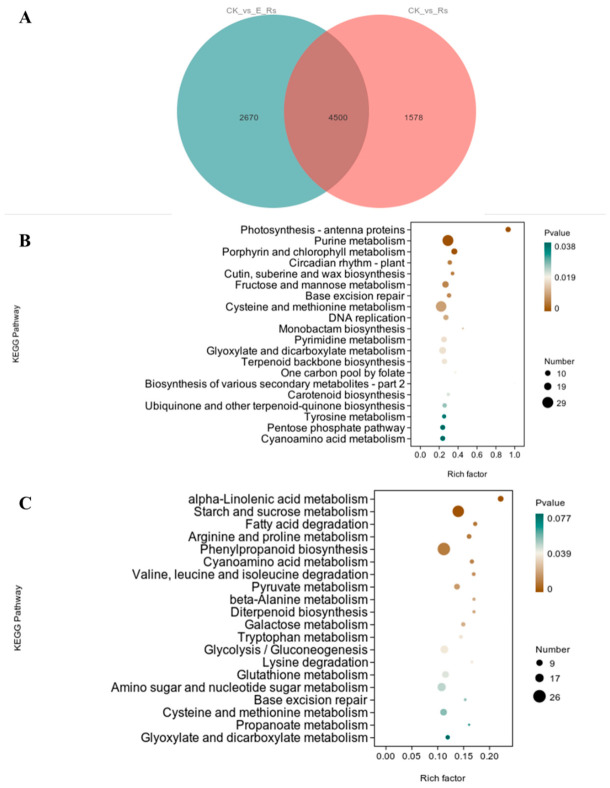
Functional enrichment analysis of DEGs. Note: (**A**) Venn plot depicting the intersection of DEGs between CK vs. Rs groups and CK vs. E_Rs groups. (**B**,**C**) KEGG pathway analysis of Gene Set A (**B**) and Gene Set B (**C**).

To elucidate the effects of (E)-2-hexenal treatment on rice resistance to *R. solani*, a comparative analysis of DEGs between CK vs. Rs and CK vs. E_Rs groups was carried out. Venn analysis identified 4500 common DEGs at the intersection of the two comparison groups (Supplementary Table S6), indicating their crucial roles in rice systemic defense mechanisms against *R. solani* infection, regardless of (E)-2-hexenal treatment. Meanwhile, 2670 unique DEGs identified in the CK vs. E_Rs comparison (Supplementary Table S7) are likely involved in mediating the rice pathogen response mechanisms specifically enhanced by (E)-2-hexenal treatment (Figure 11A).

To investigate the association between (E)-2-hexenal treatment and alterations in gene expression in rice leaves following *R. solani* infection, and to elucidate the metabolic pathways involving DEGs, we conducted KEGG analysis on Gene Sets A and B. For Gene Set A, the DEGs were significantly enriched in pathways including purine metabolism, porphyrin and chlorophyll metabolism, fructose and mannose metabolism, and cysteine and methionine metabolism (Figure 11B). In contrast, Gene Set B exhibited significant enrichment in alpha-linolenic acid metabolism, phenylpropanoid biosynthesis, starch and sucrose metabolism, diterpenoid biosynthesis, and pyruvate metabolism (Figure 11C). These findings collectively suggest that (E)-2-hexenal treatment likely modulates rice hormone signaling pathways, particularly the expression of JA and SA, through the enhancement of alpha-linolenic acid metabolism and phenylpropanoid biosynthesis. Such adaptive metabolic reprogramming may facilitate rice systemic resistance mechanisms against *R. solani* infection.

#### 3.7.5. Identification of Key Candidate Genes

Following *R. solani* infection, rice plants treated with (E)-2-hexenal exhibited significant upregulation of genes associated with defense signaling pathways, including phenylalanine ammonia-lyase genes *OsPALs*, jasmonate ZIM-domain proteins *OsJAZs*, pyrabactin resistance-like ABA receptors *OsPYLs*, pathogenesis-related proteins *OsPRs*, 12-oxophytodienoate reductase genes *OsOPRs*, WRKY transcription factors *OsWRKYs*, stress-activated protein kinases *OsSAPKs*, and NADPH oxidase genes *OsRbohs*. Together, these results indicate that building upon these findings, it can be inferred that (E)-2-hexenal treatment activates transcriptional reprogramming of these defense-related genes, suggesting that they may serve as key candidate genes in the rice response to *R. solani* infection.

### 3.8. Identification of Disease Resistance of OsRbohB Overexpression and Knockout Mutant Plants

**Figure 12:**
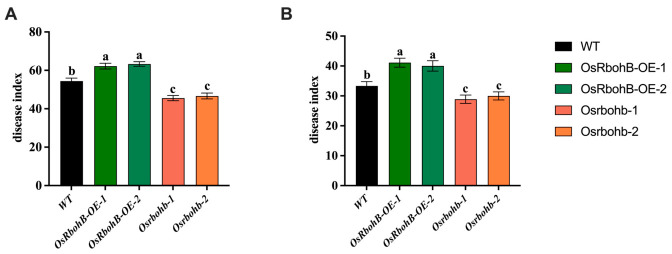
Identification of disease resistance of *OsRbohB* overexpression and knockout mutant plants. Note: (**A**) Disease index of *OsRbohB* overexpression and knockout mutant plants after infection by *R. solani*; (**B**) The disease index and resistance induction effect of *OsRbohB* overexpression and knockout mutant plants infected by *R. solani* after treatment (E)-2-hexenal. The data is expressed as mean ± standard error (N = 15). Statistical analysis between the two groups was performed using Student’s *t*-test. Different letters indicate (a, b, c) significant differences at the *p* < 0.05 level.

To provide more accurate data, we have used *OsRbohB* overexpression and knockout mutant plants to identify disease resistance. We found that when *R. solani* infected the knockout mutant plants of *osrbohb*, their disease index was significantly lower than that of WT and overexpression lines of *OsRbohB*, and the inducing resistance effect increased. The disease index of the *OsRbohB* overexpression strains increased, which were 62.22 and 63.33 respectively; the knockout of mutant plants of *osrbohb* could reduce the disease index of rice sheath blight to 45.56 and 46.67 respectively (Figure 12A). It is indicated that *OsRbohB* can affect the infection of *R. solani* and plays a negative regulatory role in the resistance of rice to *R. solani* infection. Induction treatment with (E)-2-hexenal could reduce the disease index of *R. solani*. The disease index of sheath blight in the knockout mutant plants treated with (E)-2-hexenal was higher than that of WT, which was 28.89 and 30 respectively. The disease index of the overexpression lines was significantly higher than that of the WT, which were 41.11 and 40 respectively. (E)-2-hexenal can induce rice to develop resistance to sheath blight (Figure 12B).

## 4. Discussion

It has been found that many VOCs released by almost all plants have antimicrobial, bacteriostatic, and fungicidal properties [57]. (E)-2-hexenal has been demonstrated to exhibit significant fungistatic activity, effectively inhibiting the growth of various pathogens, such as storage-associated fungi pathogens *A. flavus*, *A. niger*, and *F. graminearum* [28]. In our research, the inhibitory effect of six representative HIPVs produced by pests infesting rice on *R. solani* was determined by the mycelial growth rate method. Pathogen bioassays demonstrated that (E)-2-hexenal exposure reduced susceptibility to *R. solani*, suggesting a direct inhibitory effect of (E)-2-hexenal on *R. solani*. Furthermore, (E)-2-hexenal has a strong inhibitory effect on citrus (*Citrus reticulata*) acid rot pathogen *Geotrichum citri-aurantii* and could disrupt the integrity of the cell wall of the fungus, with changes in mycelial morphology such as dimpling, shriveling and irregular twisting [58]. In this study, it was found that (E)-2-hexenal directly suppressed the hypha growth of *R. solani* by causing contraction of the hyphal cell wall and a reduction in chitinase activity.

Previous research has substantiated that exogenous application of VOCs can induce disease resistance in various plant species. For example, one study demonstrated that exogenous (E)-2-hexenal treatment resulted in a significant reduction in the ability of *Penicillium extensum* to infect pears (*Pyrus* spp.) and apples [59]. However, the mechanism underlying the inhibitory effect of (E)-2-hexenal on rice sheath blight, as well as its molecular mechanism of inducing disease resistance in rice, remain unclear and require systematic investigation. In this study, the detached leaf bioassay showed that (E)-2-hexenal yielded a marked protective effect on rice leaves, and could effectively inhibit the invasion and expansion of *R. solani* on rice leaves; the whole-plant inoculation bioassay showed that pre-spraying (E)-2-hexenal could significantly reduce the symptoms of *R. solani* infestation and lower the disease index, indicating that (E)-2-hexenal could induce rice resistance against *R. solani*.

Priming in most cases manifests as an increase in defense responses as a result of treatment with the inducing agent, followed by a larger increase in the induced tissues after pathogen inoculation. However, the establishment of an induced resistance phenotype often necessitates a reallocation of resources from growth to defense, which can lead to a temporary growth penalty [60]. Studies have shown that (E)-2-hexenal adversely affects plant root morphogenesis by inhibiting the growth and development of plant roots [61,62]. These results suggest that (E)-2-hexenal exposure prompts plants to temporarily divert resources from growth to immune responses; this physiological trade-off may explain the attenuated priming effect observed at elevated (E)-2-hexenal concentrations, as shown in Figure 5. However, current evidence suggests that these reallocation costs do not persist long term. Similarly, VOCs exposure in very young maize seedlings only reduced seedling growth rates solely during the initial 24 h period after exposure, further substantiating that allocation costs associated with VOCs exposure are transient and do not lead to long-term impairments of growth or development [63].

When infected by pathogens or stimulated by external signals, plants will activate a series of defense responses, among which the enhancement of defense enzyme activities and accumulation of resistance-related substances constitute important physiological and biochemical defense mechanisms. Studies have shown that dimethyl disulfide (DMDS) could significantly reduce disease severity caused by canker pathogens in poplar (*Populus*), promoting the accumulation of defense enzyme activities, MDA and total phenol (TP), while transcriptomic analysis indicated DMDS may induce plant disease resistance by affecting hormone signaling pathways [17]. This provides important references for this study to investigate the mechanism of (E)-2-hexenal-induced resistance in rice plants. Our research results substantiated that (E)-2-hexenal treatment could increase the activities of disease-resistant enzymes and H_2_O_2_ content in rice leaves. Experiments also found that (E)-2-hexenal treatment can increase the content of disease-resistant secondary metabolites, such as flavonoids and AA. It was found that (E)-2-hexenal may activate the antioxidant defense system of rice, and alleviate oxidative damage caused by pathogen infection, indicating that (E)-2-hexenal may induce SAR in rice through ROS signaling transduction pathways, thereby enhancing rice resistance against sheath blight.

The disease resistance response in rice involves a series of plant hormones in biotic stress signaling, such as JA and SA. This is based on the classical model established in *A. thaliana*, which posits that SA mediates defense against biotrophic pathogens while JA counters necrotrophic pathogens and herbivores [34]. However, numerous exceptions to this general principle have been reported in *A. thaliana* and dicots, and in rice, the dichotomy appears to be non-existent [64]. Instead of viewing SA and JA as co-equal and often antagonistic regulators of distinct immune pathways, JA emerges as the principal regulator of immunity in rice, with SA playing a subordinate role [64]. JA metabolism has also been implicated in induced resistance against *R. solani* in rice [65], and exogenous JA application has been shown to significantly enhance rice resistance to *Xanthomonas oryzae* [66,67]. In rice, the significance of the SA-signaling pathway in defense responses remains subject to debate, based on observations that rice contains high levels of SA even without pathogen infection, and that SA levels in rice do not appear to increase in response to pathogen infection [68]. However, recent studies have shown that the SA-signaling pathway plays a crucial role in the defense responses in rice [69]. Notably, the crosstalk between volatiles and other plant hormones and stress factors constitutes an extremely complex regulatory network [36]. HPLC-MS was conducted to reveal significant changes in hormone levels, with enrichment of JA and SA. This suggested that the mechanism of (E)-2-hexenal-induced resistance to rice sheath blight may be related to hormone-mediated SAR in rice plants.

The activation of plant immune systems involves complex regulatory signaling networks, where calcium ions, ROS, mitogen-activated protein kinase (MAPK) cascades, and phytohormone signaling molecules play pivotal roles in inducing plant defense responses [70]. The establishment of SAR is accompanied by extensive reprogramming of plant gene expression. Transcriptomic analysis serves as a crucial approach for investigating gene expression changes. Our transcriptome analysis revealed that (E)-2-hexenal treatment following *R. solani* infection significantly affected multiple metabolic pathways in rice, particularly by regulating α-linolenic acid metabolism, diterpenoid biosynthesis, phenylpropanoid biosynthesis, and plant hormone signal transduction pathways to promote the biosynthesis of hormones such as SA and JA. These findings corroborate previous hormone quantification results. Concurrently, transcriptomic analysis also identified the activation of several disease resistance-related signaling pathways, including MAPK signaling pathway-plants, plant–pathogen interaction, and secondary metabolite biosynthesis pathways. Pathogenesis-related (PR) genes, SAR-related genes, and plant defensin genes were induced to be expressed, and the expressions of transcription factors were changed. These results suggested that (E)-2-hexenal may induce rice resistance against rice sheath blight through activation of these signaling pathways.

As one of the primary pathways for ROS production in plants, Rboh plays a significant role in plant resistance to stress. ROS production is a critical signaling response in combating pest and pathogen attacks. Investigating the functional mechanisms of Rboh in plant responses to pest and pathogen attacks will contribute to enhancing plant production potential. ROS signaling mediated by Rboh serves as a key regulatory hub in plants and is interconnected with numerous stress response signals. Transcriptome analysis in this chapter indicates that after rice is inoculated with *R. solani*, the NADPH oxidase gene *OsRbohB* may play an important role in combating rice sheath blight. Disease resistance evaluation of *OsRbohB* overexpression and knockout mutant plants revealed that *OsRbohB* acts as a negative regulator in rice sheath blight resistance. Overexpression of *OsRbohB* significantly enhanced the infection behavior of *R. solani* in rice, while knocking out *OsRbohB* significantly improved rice resistance to *R. solani*. However, its function in sheath blight resistance has not been fully elucidated. Meanwhile, disease resistance evaluation results showed that (E)-2-hexenal treatment significantly reduced the disease index, suggesting that (E)-2-hexenal may enhance resistance by regulating OsRbohB-mediated ROS signaling.

Overall, our study elucidates the mechanism of the rice plant defense response to (E)-2-hexenal to some extent and provides novel insights into the mechanism of resistance conferred by (E)-2-hexenal against rice sheath blight. However, several limitations should be acknowledged. Notably, our conclusions regarding the functional role of specific genes or pathways were primarily derived from transcriptomic analyses and phenotypic observations. Although these approaches revealed significant correlations between gene expression and phenotypic traits, there were an absence of direct genetic evidence through stable transformants or genome-edited lines. Future studies employing CRISPR/Cas9-mediated gene knockout or transgenic overexpression models would be critical to validate the hypothesized molecular mechanisms.

Taken together, our data demonstrate that (E)-2-hexenal-induced resistance against rice sheath blight likely results from multifaceted synergistic effects, involving the direct antifungal activity of (E)-2-hexenal, enhanced activities of defense-related enzymes and accumulation of antimicrobial compounds in rice, and activation of ROS signaling, SA and JA hormone signaling pathways, which collectively induce rice disease resistance against *R. solani*. In conclusion, our findings reveal a complex interaction between rice and *R. solani* following exposure to exogenous (E)-2-hexenal. Our results offer valuable insights into managing rice diseases via more environmentally friendly ways using (E)-2-hexenal, or potentially other VOCs, and further contribute to our understanding of plant defense mechanisms, ultimately leading to more effective control strategies for insect pests and plant diseases.

## Data Availability

The original contributions presented in this study are included in the article/Supplementary Material. Further inquiries can be directed to the corresponding author. Part of the RNA-seq data has been indicated in the manuscript, while the remaining part will be made available on request from the corresponding author.

## Supplementary Materials

The following supporting information can be downloaded at: https://www.mdpi.com/article/10.3390/plants15101581/s1, Table S1. Basic information of VOCs used in this study; Table S2. Statistical summary of differential genes induced by exogenous; Table S3. GO annotation classification statistics of differentially expressed genes induced by Rhizoctonia solani infection under different treatments.; Table S4. Results of KEGG annotation of differentially expressed genes induced by Rhizoctonia solani infection under different treatments; Table S5. DEGs in rice-related pathways induced by exogenous (E)-2-hexenal application and R. solani infection; Table S6. Functional annotation of genes in Gene Set A. Table S7. Functional annotation of genes in Gene Set B.
