# Supplementary material for: (E)-2-Hexenal Combats Rice Sheath Blight Through Direct Pathogen Inhibition and Host Defense Reprogramming
Source: Plants (Basel). 2026 May 21;15(10):1581. doi: 10.3390/plants15101581 (PMC13211129; doi:10.3390/plants15101581)
Supplement: Supplementary file 1 [file plants-15-01581-s001.zip › plants-4244965-supplementary.pdf]

Table S1. Basic information of VOCs used in this study.

Serial number	Substance name	CAS Registry Number	Content
1	*(E)*-2-hexenal	6728-26-3	98.0%
2	Linalool	78-70-6	98.0%
3	leaf alcohol	928-96-1	98.0%
4	3-Octanone	106-68-3	98.0%
5	trans-Caryophyllene	87-44-5	＞80.0%
6	α-Pinene	80-56-8	≥99.5%

Table S2. Statistical summary of differential genes induced by exogenous *(E)*-2-hexenal application and *R. solani* infection in rice.

DEG Set	DEG Number	up-regulated	down-regulated
CK vs E	3217	1512	1705
CK vs Rs	6078	2767	3311
CK vs E_Rs	7170	3274	3896
Rs vs E_Rs	2006	1236	770

† "CK" represents control group; "E" represents rice samples treated by *(E)*-2-hexenal; "Rs" represents rice samples infected by *R. solani*; "E_Rs" represents rice samples infected by *R. solani* after *(E)*-2-hexenal treatment.

Table S3. GO annotation classification statistics of differentially expressed genes induced by Rhizoctonia solani infection under different treatments.

GO_ID	Category	Term	Up	Down	DEG
GO:0008152	BP	metabolic process	527	279	806
GO:0110165	CC	cellular anatomical entity	477	320	797
GO:0009987	BP	cellular process	449	265	714
GO:0003824	MF	catalytic activity	466	227	693
GO:0005488	MF	binding	412	207	619
GO:0005622	CC	intracellular anatomical structure	300	186	486
GO:0050896	BP	response to stimulus	289	142	431
GO:0016020	CC	membrane	227	175	402
GO:0005737	CC	cytoplasm	246	147	393
GO:0043226	CC	organelle	230	155	385
GO:0043229	CC	intracellular organelle	230	155	385
GO:0043227	CC	membrane-bounded organelle	230	150	380
GO:0043231	CC	intracellular membrane-bounded organelle	230	150	380
GO:0044238	BP	primary metabolic process	205	148	353
GO:0071704	BP	organic substance metabolic process	205	148	353
GO:0071944	CC	cell periphery	175	129	304

GO_ID: GO Term number; Catergory: the classification in which the GO Term is located; Term: the name of the GO entry; Up/Down: the number of up/down-regulated genes enriched to the GO entry; DEG: the total number of differential genes enriched to the GO entry.

Table S4. Results of KEGG annotation of differentially expressed genes induced by Rhizoctonia solani infection under different treatments.

Pathway	Up	Down	DEG
Phenylpropanoid biosynthesis	15	6	21
Plant-pathogen interaction	18	1	19
Amino sugar and nucleotide sugar metabolism	10	8	18
Starch and sucrose metabolism	7	11	18
MAPK signaling pathway - plant	13	4	17
Plant hormone signal transduction	9	7	16
alpha-Linolenic acid metabolism	12	3	15
Glutathione metabolism	11	3	14
Diterpenoid biosynthesis	11	2	13
Glycolysis / Gluconeogenesis	7	4	11
Cyanoamino acid metabolism	3	7	10
Tryptophan metabolism	5	4	9
Pyruvate metabolism	6	3	9
Cysteine and methionine metabolism	7	2	9
Fatty acid degradation	4	4	8
Arginine and proline metabolism	6	2	8
Flavonoid biosynthesis	4	3	7
Valine, leucine and isoleucine degradation	5	2	7

Pathway: Pathway name; Up/Down: number of up/down-regulated genes enriched to the pathway; DEG: total number of differential genes enriched to the pathway.

Table S5. DEGs in rice-related pathways induced by exogenous (E)-2-hexenal application and *R. solani* infection.

	Gene name	Pathway	Log2FC
LOC_Os03g08320	OsJAZ11	Plant hormone signal transduction	1.1469
LOC_Os10g25290	OsJAZ12	Plant hormone signal transduction	1.3874
LOC_Os10g25230	OsJAZ13	Plant hormone signal transduction	1.6876
LOC_Os01g28450	OsPR1b	Plant-pathogen interaction	1.5007
LOC_Os01g61080	OsWRKY24	MAPK signaling pathway - plant	1.4881
LOC_Os05g39720	OsWRKY70	MAPK signaling pathway - plant	1.7165
LOC_Os06g51050	OsCHI11	MAPK signaling pathway - plant	2.3151
LOC_Os05g35290	OsPAL7	Phenylpropanoid biosynthesis	1.2574
LOC_Os06g11290	OsOPR1	alpha-Linolenic acid metabolism	2.004
LOC_Os03g12500	OsAOS2	alpha-Linolenic acid metabolism	1.0856

Table S6. Functional annotation of genes in Gene Set A.

Gene ID	Gene expression levels (fpkm)				Gene symbol
	CK	E	Rs	E_Rs	
LOC_Os02g41650	31.5733	16.59774	66.64747	65.1507	*OsPAL2*
LOC_Os02g15640	45.87761	66.13372	72.853	73.74246	*OsPYL3*
LOC_Os06g36670	0.257914	0.083042	1.430067	1.134142	*OsPYL9*
LOC_Os03g18600	11.36071	19.37045	21.22025	25.27686	*OsPYL4*
LOC_Os09g28310	27.89203	13.50144	49.71029	41.58139	*OsbZIP72*
LOC_Os02g13330	0.361452	0.184371	0.697025	0.634171	*OsPYL2*
LOC_Os12g05680	0.521043	0.610689	1.170315	0.765211	*OsbZIP83*
LOC_Os12g39630	56.10464	76.41893	0.724064	0.806542	*OsSAPK9*
LOC_Os01g46760	15.11376	4.009867	0.4938	0.195156	*OsPP2C8*
LOC_Os04g35240	18.024	8.653107	1.90915	1.304751	*OsSAPK7*
LOC_Os03g16170	12.691	4.577992	4.373124	3.158284	*OsPP2C30*
LOC_Os09g26780	54.46419	11.73682	11.74533	8.089396	*OsJAZ8*
LOC_Os07g48660	0.420806	0.352631	0.025159	0.069429	*OsbZIP62*
LOC_Os07g03710	5.74745	8.092197	1.459572	1.077732	*OsPR1a*
LOC_Os05g41280	0.474156	0.225224	0	0.02576	*OsbZIP43*
LOC_Os02g35310	9.205875	7.633075	1.695462	1.08611	*OsOPR8*
LOC_Os01g25820	50.03238	57.41695	10.0154	6.09362	*OsRbohB*
LOC_Os12g35610	0.041779	0.046571	0	0.001127	*OsrbohH*
LOC_Os12g12860	0.431355	0.498219	0.053664	0.017628	*OsCPK29*
LOC_Os11g07040	40.46788	35.50639	11.0001	13.69366	*OsCDPK14*
LOC_Os08g38990	7.398449	21.7722	1.932628	1.593159	*OsWRKY30*

Table S7. Functional annotation of genes in Gene Set B.

Gene ID	Gene expression levels (fpkm)				Gene symbol
	CK	E	Rs	E_Rs	
LOC_Os12g33610	17.99313	10.67845	79.21562	81.72896	OsPAL9
LOC_Os05g35290	6.085805	2.636239	9.18524	21.99341	OsPAL7
LOC_Os02g41670	0.979574	0.468676	0.939409	4.654425	OsPAL3
LOC_Os02g41680	6.567497	2.170767	13.76554	15.22967	OsPAL4
LOC_Os09g23660	12.64543	8.103483	15.11489	21.79864	OsJAZ4
LOC_Os01g59350	5.027803	23.33071	6.750322	9.22532	OsbZIP28
LOC_Os07g48820	42.4634	37.56201	61.8614	68.41147	OsbZIP63
LOC_Os01g64730	15.09731	24.07384	19.05657	25.35159	OsbZIP12
LOC_Os02g52780	12.20678	9.42218	17.25499	19.90185	OsbZIP23
LOC_Os05g39580	0.196568	0.521825	0.162794	0.54627	OsPYL5
LOC_Os07g03730	54.47092	43.28157	60.76054	226.4752	OsPR1aL
LOC_Os03g20650	1.308985	0.612848	0.644561	0.145591	OsbZIP29
LOC_Os06g11290	4.824588	5.855158	3.518812	13.58092	OsOPR1
LOC_Os03g12500	3.720719	6.846044	3.035511	6.414412	OsAOS2
LOC_Os12g37260	409.6457	201.1704	151.2737	37.65257	OsLOX11
LOC_Os11g33120	34.80477	41.23732	45.0834	57.71927	OsrbohI
LOC_Os06g51050	3.105538	3.145908	1.254884	6.217065	OsCHI11
LOC_Os07g38120	17.68498	18.17102	28.91624	37.64987	OsCPK20
LOC_Os09g30400	32.11682	38.83969	37.99838	52.1635	OsWRKY8
